# Supplementary material for: Molecule-based nonlinear optical switch with highly tunable on-off temperature using a dual solid solution approach
Source: Nat Commun. 2020 Jun 2;11:2752. doi: 10.1038/s41467-020-15518-z (PMC7265397; doi:10.1038/s41467-020-15518-z)
Supplement: Supplementary file 1 — Supplementary Information [file 41467_2020_15518_MOESM1_ESM.pdf]

Supplementary Information for

Molecule-based nonlinear optical switch with highly tunable on-off temperature using a dual solid solution approach

Zhang et al.

Supplementary Table 1 Summary of crystal data and structural refinement for CPs **1** and **2**

Compound	**1**	**1**	**2**
T (K)	296	348	296
Phase	**1α**	**1β**	**2α**
Empirical formula	$C_8H_{14}N_4S_3Cd_1$		
Formula weight	374.81		
Space group	$Cmc2_1$	$P6_3/mmc$	$P2_1/c$
a (Å)	9.3607(1)	9.0967(14)	10.8743(3)
b (Å)	14.6857(2)	9.0967(14)	14.7852(4)
c (Å)	10.6812(1)	10.7071(10)	18.5830(6)
β (deg)	90	90	106.892(2)
V (Å^3)	1468.33(3)	767.31(18)	2858.84(14)
Z	4	2	4
D_{calcd} (g cm^{-3})	1.696	1.622	1.742
M (mm^{-1})	1.895	1.813	1.946
GOF on F^2	1.052	1.127	1.028
$R1$, $wR2$ [I>2σ(I)]^a	0.0177, 0.0397	0.0487, 0.1111	0.0399, 0.1086
$R1$, $wR2$ (all data)	0.0194, 0.0406	0.1143, 0.1394	0.0549, 0.1195

$^aR_1 = \Sigma||F_o| - |F_c||/\Sigma|F_o|$, $wR_2 = \{\Sigma w[(F_o)^2 - (F_c)^2]^2/\Sigma w[(F_o)^2]^2\}^{1/2}$

Supplementary Table 2 Selected bond lengths (Å) for CP **1** at two different temperatures

296 K			
Cd(1)–N(1)	2.270(3)	Cd(1)–N(2)#1	2.331(2)
Cd(1)–S(1)#1	2.7328(9)	Cd(1)–S(2)	2.7457(6)
348 K			
Cd(1)–N(1)#2	2.29(1)	Cd(1)–S(1)#3	2.74(1)

Symmetry codes: #1 $-x + 2, -y, z - 1/2$; #2 $x - y, x, -z + 2$; #3 $-y, x - y, -z + 3/2$.

Supplementary Table 3 Selected bond lengths (Å) for CP **2** at 296 K

Cd(1)–N(1)	2.263(4)	Cd(1)–N(4)#1	2.318(4)
Cd(1)–N(6)#1	2.360(4)	Cd(1)–S(2)	2.7093(13)
Cd(1)–S(5)#1	2.7462(13)	Cd(1)–S(3)	2.7805(12)
Cd(2)–N(5)	2.263(4)	Cd(2)–N(2)	2.326(4)
Cd(2)–N(3)	2.328(4)	Cd(2)–S(6)	2.7075(12)

Cd(2)–S(1)	2.7409(14)	Cd(2)–S(4)	2.7949(13)
S(1)–C(1)	1.634(4)	S(2)–C(2)	1.634(4)
S(3)–C(3)	1.636(4)	S(4)–C(4)	1.628(4)
S(5)–C(5)	1.639(4)	S(6)–C(6)	1.643(4)
N(1)–C(1)	1.155(5)	N(2)–C(2)	1.147(5)
N(3)–C(3)	1.155(5)	N(4)–C(4)	1.149(5)
N(5)–C(5)	1.149(5)	N(6)–C(6)	1.150(5)

Symmetry codes: #1 *x* + 1, *y*, *z*.

Supplementary Table 4 EXAFS fitting parameters at the Cd *K*-edge for CPs **1** and **2** at different temperatures. $Cd_{(aq)}$ and CdS are used as the reference samples (S_0^2 = 0.839)

Sample	Shell	^aN	^bR (Å)	$^c\sigma^2$ (Å^2)	$^d\Delta E_0$ (eV)	^eR factor
$Cd_{(aq)}$	Cd–O	6.0	2.27	0.0082	1.2	0.0007
CdS	Cd–S	4.0	2.53	0.0046	0.5	0.0033
**1** at 296 K	Cd–N	2.9	2.26	0.0080	–2.6	0.0001
	Cd–S	3.0	2.72	0.0147		
**1** at 353 K	Cd–N	2.7	2.25	0.0088	–2.6	0.0003
	Cd–S	2.8	2.70	0.0163		
**1** at 398 K	Cd–N	2.8	2.21	0.0148	–4.6	0.0005
	Cd–S	2.9	2.59	0.0191		
**2** at 296 K	Cd–N	2.8	2.25	0.0074	–4.7	0.0002
	Cd–S	2.9	2.71	0.0148		
**2** at 356 K	Cd–N	2.8	2.24	0.0097	–5.1	0.0008
	Cd–S	2.8	2.68	0.0174		
**2** at 391 K	Cd–N	2.8	2.22	0.0141	–4.0	0.0004
	Cd–S	2.9	2.59	0.0190		

^aN: coordination numbers; ^bR: bond distance; $^c\sigma^2$: Debye-Waller factors; $^d\Delta E_0$: the inner potential correction; ^eR factor: goodness of fit. S_0^2 was set to 0.839, according to the experimental EXAFS fit of $Cd_{(aq)}$ by fixing CN as the known crystallographic value.

Supplementary Table 5 Unit cell parameters of the solid solutions of CPs **1** and **2** at 296 K deduced from Pawley refinements

Compound	{$\mathbf{1}_{0.99}\mathbf{2}_{0.01}$}	{$\mathbf{1}_{0.976}\mathbf{2}_{0.024}$}	{$\mathbf{1}_{0.952}\mathbf{2}_{0.048}$}	{$\mathbf{1}_{0.667}\mathbf{2}_{0.333}$}	{$\mathbf{1}_{0.50}\mathbf{2}_{0.50}$}
Space group	$Cmc2_1$	$Cmc2_1$	$Cmc2_1$	$P6_3/mmc$	$P6_3/mmc$
a (Å)	9.3522(4)	9.3516(3)	9.3517(3)	8.9929(5)	8.9792(9)
b (Å)	14.6946(6)	14.7033(5)	14.7324(5)	8.9929(5)	8.9792(9)
c (Å)	10.6768(4)	10.6797(4)	10.6879(4)	10.6927(6)	10.694(1)

V (Å^3)	1467.28(5)	1468.46(4)	1472.49(4)	748.88(5)	746.70(1)
Residuals R_p, R_{wp}	3.32%, 4.53%	3.41%, 4.59%	3.22%, 4.60%	3.72%, 5.09%	4.32%, 6.07%

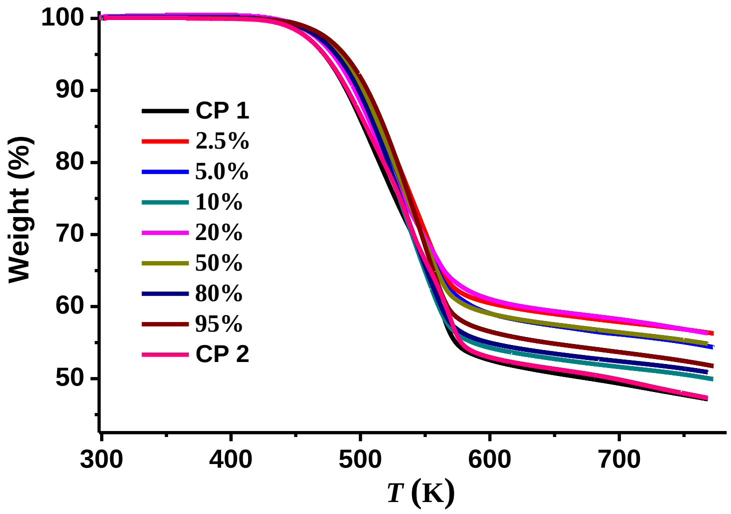

Supplementary Fig. 1 TGA curves for CPs **1**, **2**, and their solid solutions. The mixing ratio of CP **2** in these solid solutions increases from 2.5% to 95%.

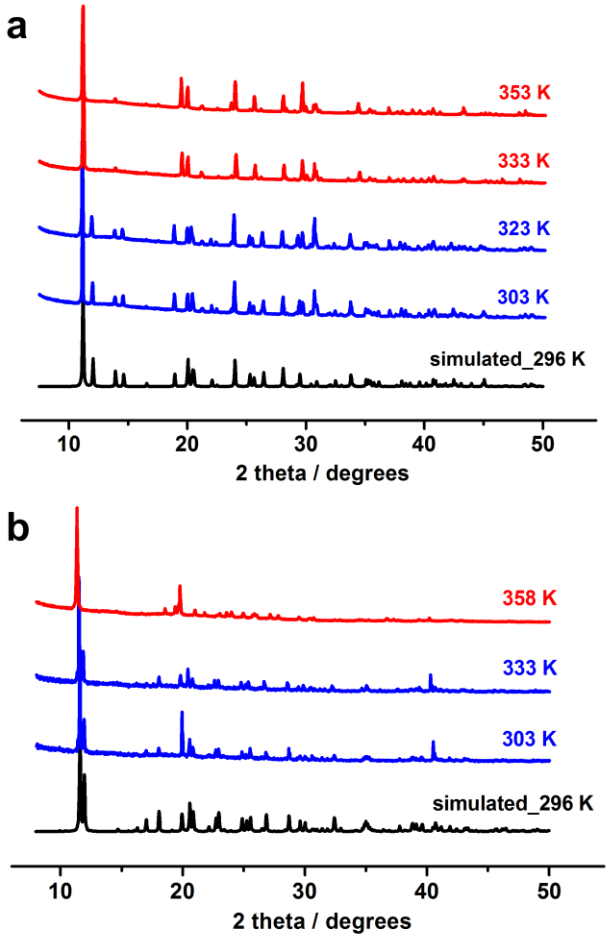

Supplementary Fig. 2 Variable-temperature powder XRD patterns of CPs **1** (**a**) and **2** (**b**).

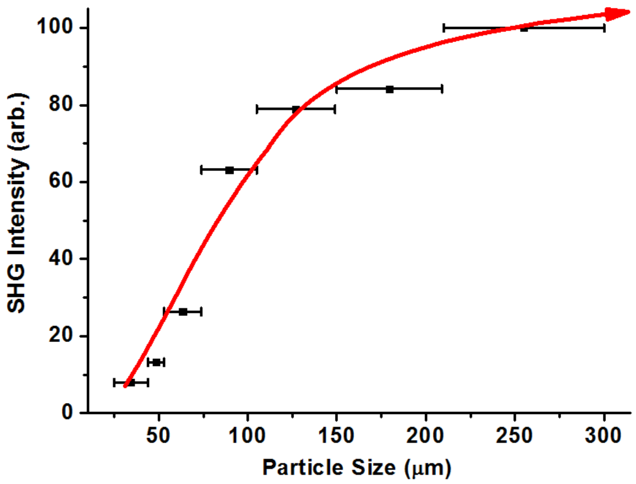

Supplementary Fig. 3 The phase matching curve for CP **1** at 296 K. The red curve is to guide the eye and is not a fit to the data (SHG intensities of **1** converted from photo-effects as a function of the particle size. The curve shows that SHG signals gradually increase to be saturated as the particle size becomes larger, which reveals the potentials of **1** as the bulk NLO materials).

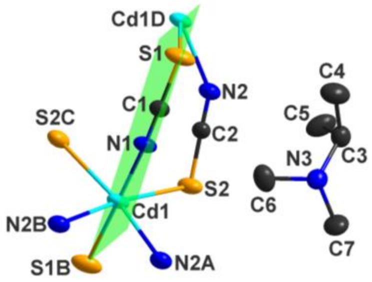

Supplementary Fig. 4 ORTEP representation of the selected unit of CP **1** at 296 K. The anisotropic displacement ellipsoids are drawn at 30% probability. Only one orientation was shown for the disordered (i-PrNHMe$_2$)$^+$ cation for clarity. The mirror plane is shaded in green. Symmetry codes for the generated atoms: A. *x*, –*y*, –1/2 + *z*; B. 2 – *x*, –*y*, –1/2 + *z*; C. 2 – *x*, *y*, *z*; D. 2 – *x*, –*y*, 1/2 + *z*.

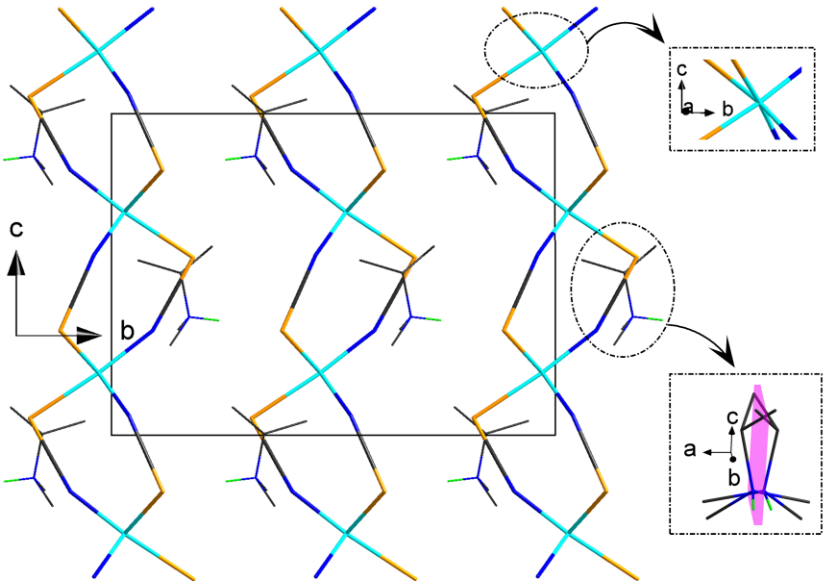

Supplementary Fig. 5 View of the three-dimensional packing structure of CP **1** down the *a*-axis at 296 K. For display details, see Fig. 5. The mirror plane is shaded in purple.

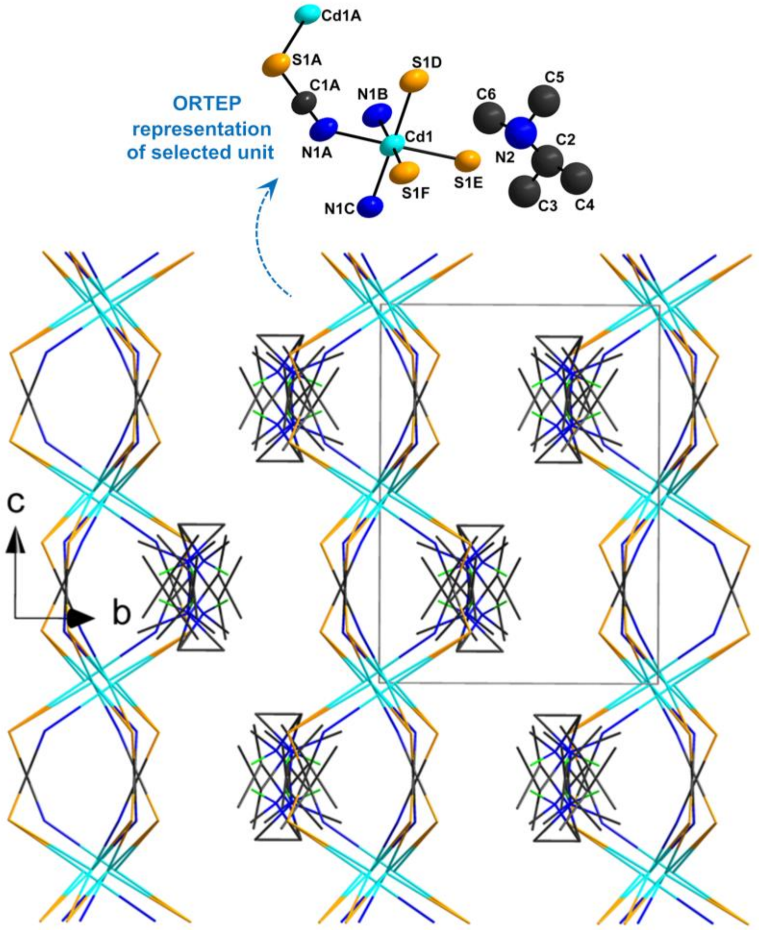

Supplementary Fig. 6 View of the three-dimensional packing structure of CP **1** down the *a*-axis at 348 K. The anisotropic displacement ellipsoids of selected unit are drawn at 30% probability. The quite disordered (i-PrNHMe$_2$)$^+$ cation is refined isotropically. Symmetry codes: A. $-x$, $-y$, $2-z$; B. $x-y$, x, $2-z$; C. $-y$, $x-$ y, z; D. $x-y$, $-y$, $1/2+z$; E. x, $x-y$, $3/2-z$; F. $-x+y$, $-x$, $3/2-z$.

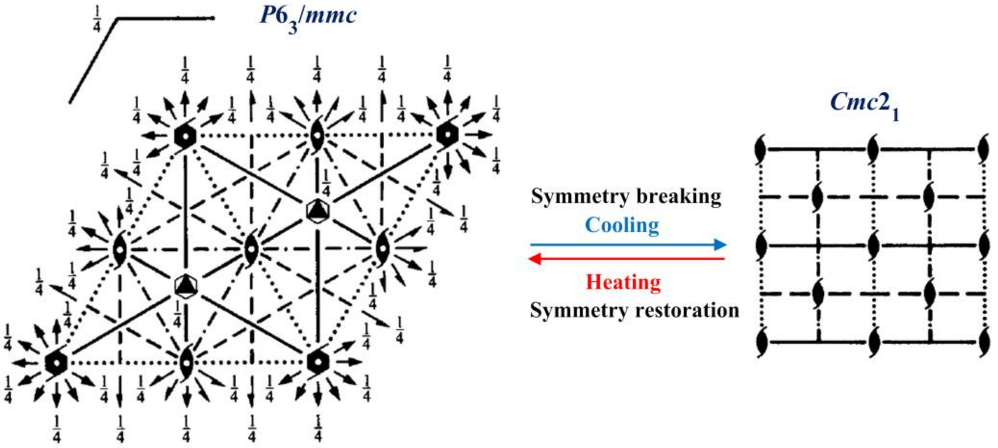

Supplementary Fig. 7 Symmetry breaking process in CP **1**. Symmetry operations for $P6_3/mmc$: (1) 1; (2) 3^+ $0,0,z$; (3) 3^- $0,0,z$; (4) $2(0,0,1/2)$ $0,0,z$; (5) 6^- $(0,0,1/2)$ $0,0,z$; (6) 6^+ $(0,0,1/2)$ $0,0,z$; (7) 2 $x,x,0$; (8) 2 $x,0,0$; (9) 2 $0,y,0$; (10) 2 $x,\bar{x},1/4$; (11) 2 $x,2x,1/4$; (12) 2 $2x,x,1/4$; (13) $\bar{1}$ $0,0,0$; (14) $\bar{3}^+$ $0,0,z$; $0,0,0$; (15) $\bar{3}^-$ $0,0,z$; $0,0,0$; (16) m $x,y,1/4$; (17) $\bar{6}^-$ $0,0,z$; $0,0,1/4$; (18) $\bar{6}^+$ $0,0,z$; $0,0,1/4$; (19) m $x,\bar{x},z$; (20) m $x,2x,z$; (21) m $2x,x,z$; (22) c x,x,z; (23) c $x,0,z$; (24) c $0,y,z$. Symmetry operations for $Cmc2_1$: (1) 1; (2) $2(0,0,1/2)$ $0,0,z$; (3) c $x,0,z$; (4) m $0,y,z$.

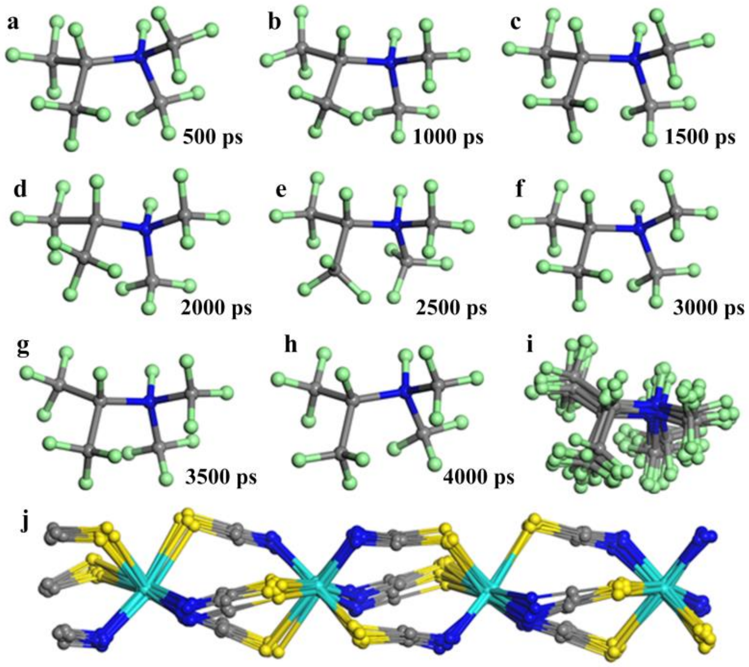

Supplementary Fig. 8 Constant-volume & temperature dynamic simulation for CP **1** at 296 K. **a-h** Local snapshots of a (i-PrNHMe$_2$)$^+$ cation over the simulation time, showing the orientation changes. **i** Overlapping maps of the snapshots of (**a**)-(**h**). **j** The corresponding local snapshots of a {[Cd(SCN)$_3$]$^-$}$_\infty$ chain are also overlapped, to display its dynamics.

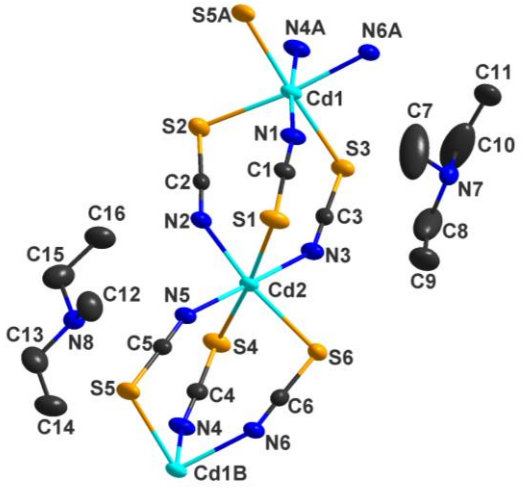

Supplementary Fig. 9 ORTEP representation of the selected unit of CP **2** at 296 K. The anisotropic displacement ellipsoids are drawn at 30% probability. Symmetry codes for the generated atoms: A. 1 + x, y, z; B. –1 + x, y, z.

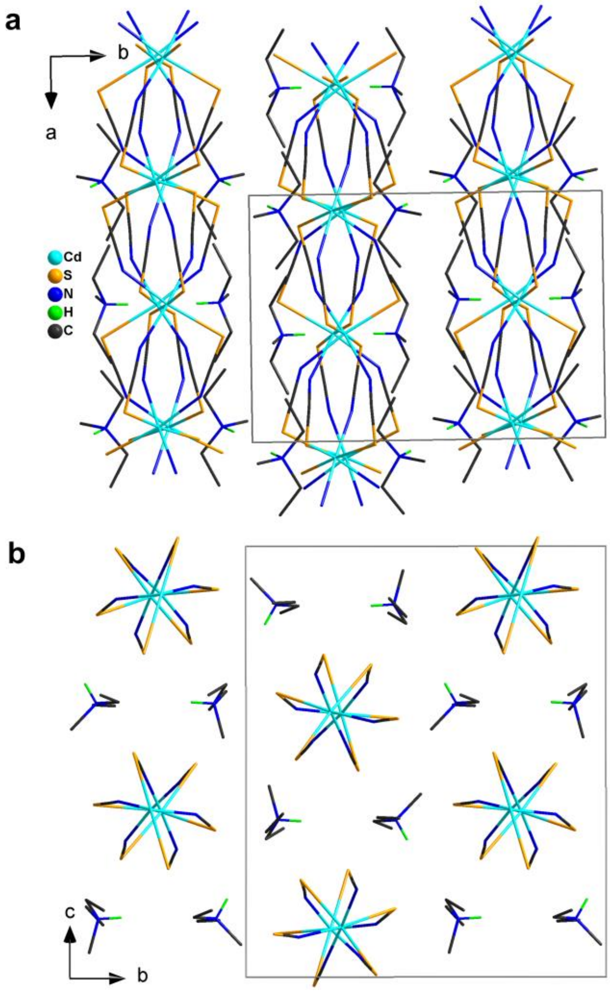

Supplementary Fig. 10 View of the three-dimensional packing structure of CP **2** down the *c*-axis (a) and *a*-axis (b), respectively. C-bound H atoms have been omitted for clarity. For display details, see Fig. 5.

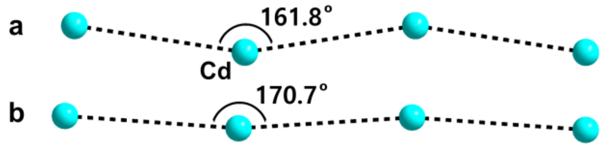

Supplementary Fig. 11 The bending degrees of the $\{[Cd(SCN)_3]^-\}_\infty$ chain in **1α** (**a**) and **2α** (**b**), characterized by the different Cd···Cd···Cd angles.

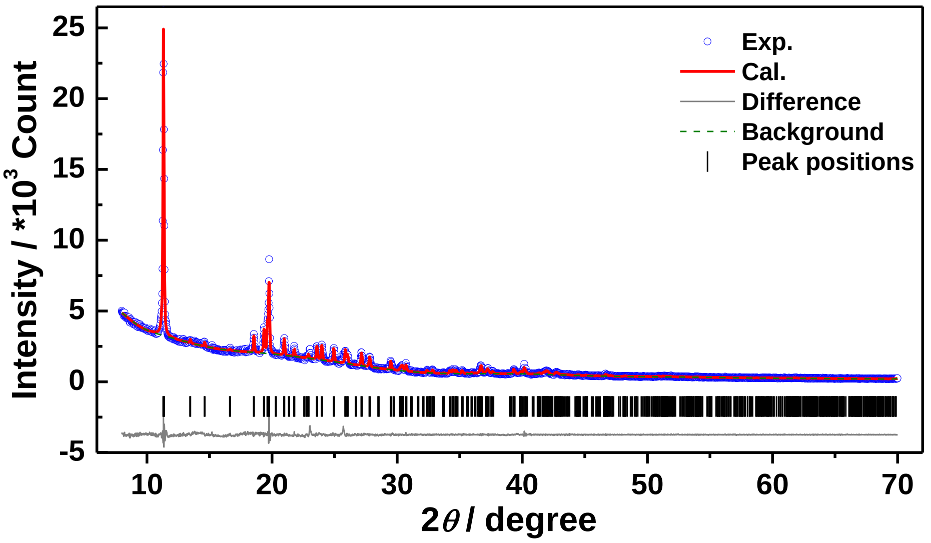

Supplementary Fig. 12 Pawley refinement on the PXRD pattern of CP **2** at 358 K. Experimental pattern (blue circles), calculated pattern (red line), difference profile (grey line) and background profile (dashed line). Stick marks (|) at the bottom of the pattern indicate peak positions allowed by the appointed unit-cell parameters and space group.

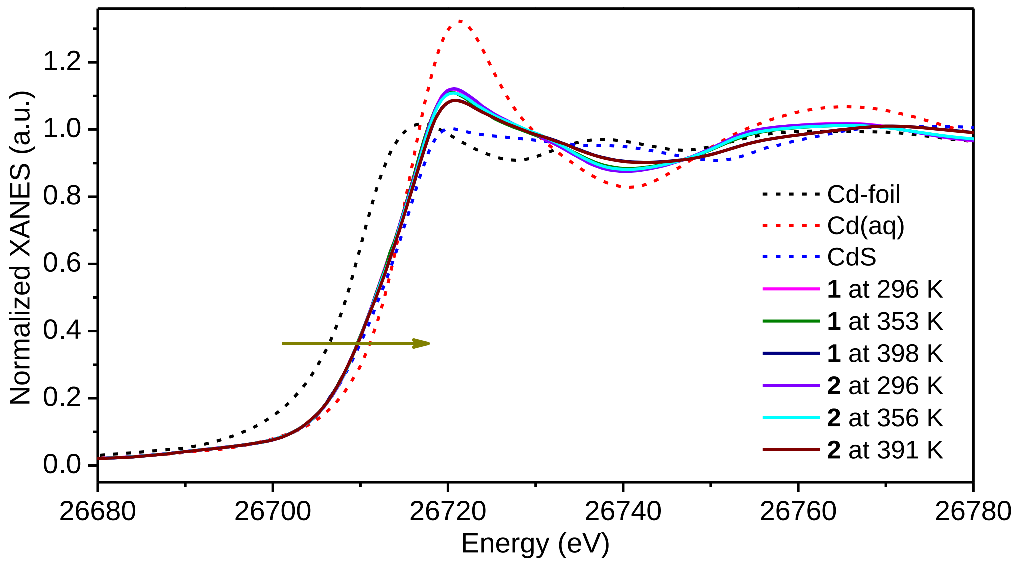

Supplementary Fig. 13 XANES at the Cd *K*-edge for CPs **1** and **2** at different temperatures. Cd foil, $Cd_{(aq)}$ and CdS are used as the reference samples.

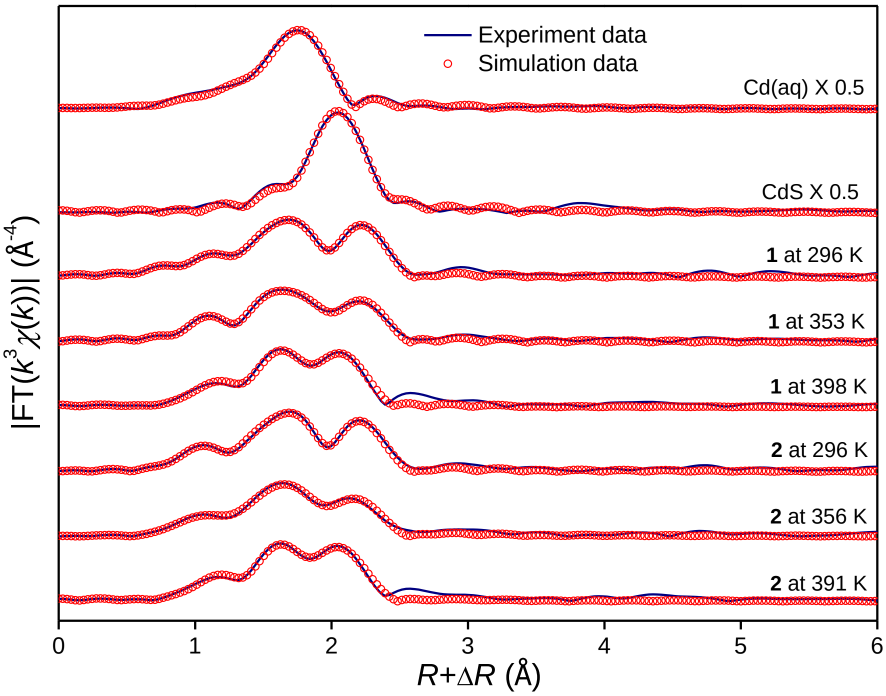

Supplementary Fig. 14 EXAFS fitting at the Cd *K*-edge for CPs **1** and **2** at different temperatures. $Cd_{(aq)}$ and CdS are used as the reference samples.

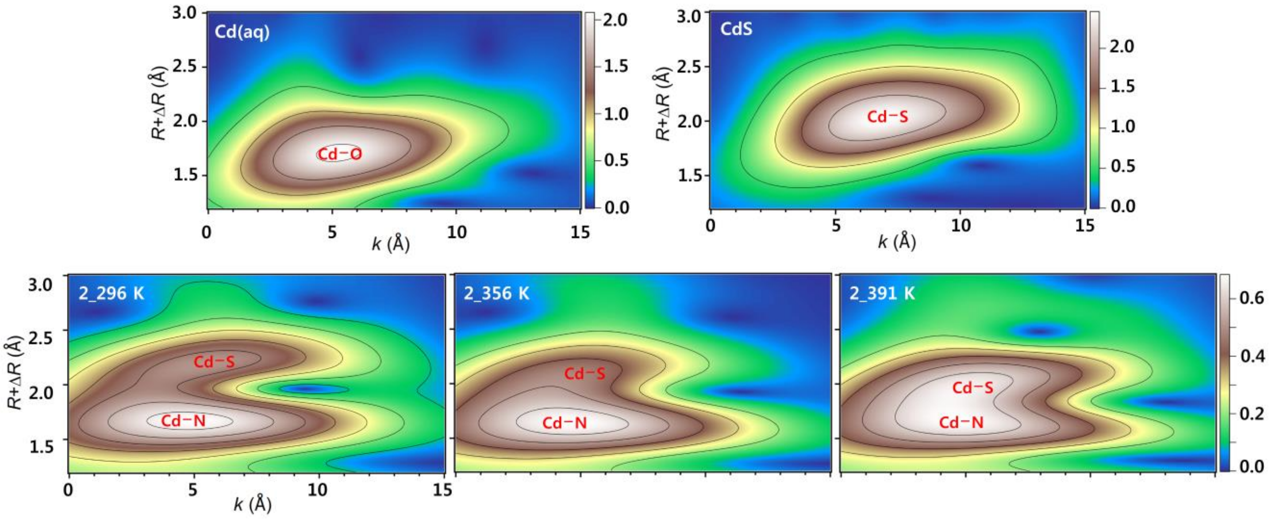

Supplementary Fig. 15 Wavelet transform EXAFS of CP **2** at different temperatures. $Cd_{(aq)}$ and CdS are used as the references. The heights of the peaks are represented by different colors.

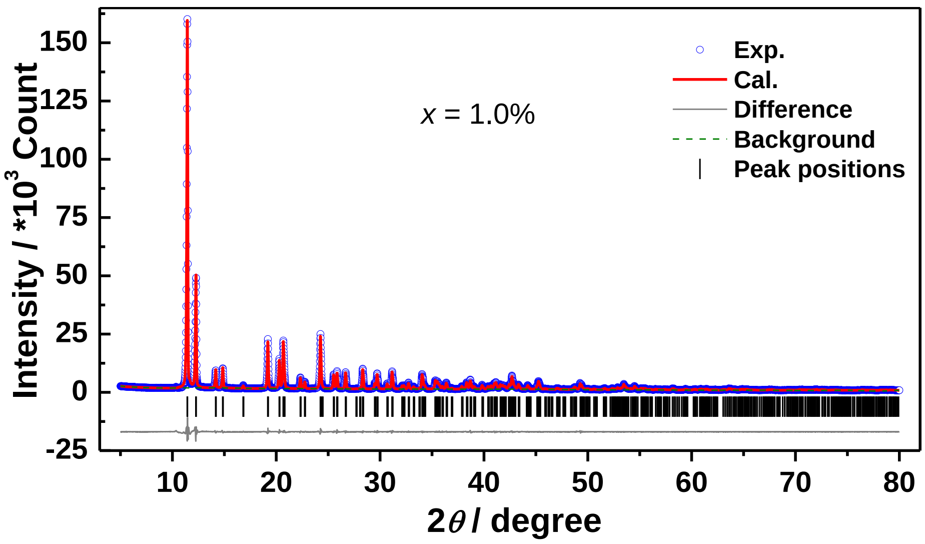
Intensity / *10^3 Count
2θ / degree
x = 1.0%
Exp.
Cal.
Difference
Background
Peak positions

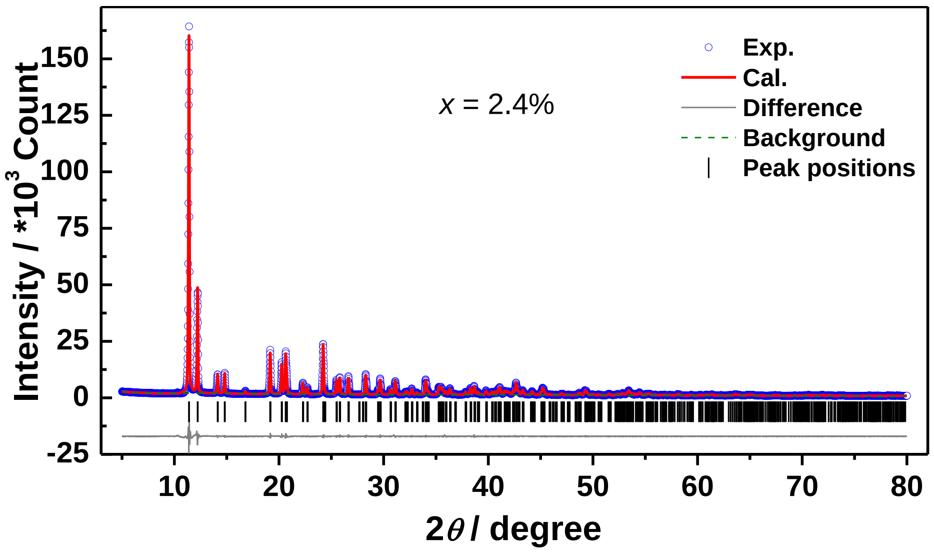
Intensity / *10^3 Count
2θ / degree
x = 2.4%
Exp.
Cal.
Difference
Background
Peak positions

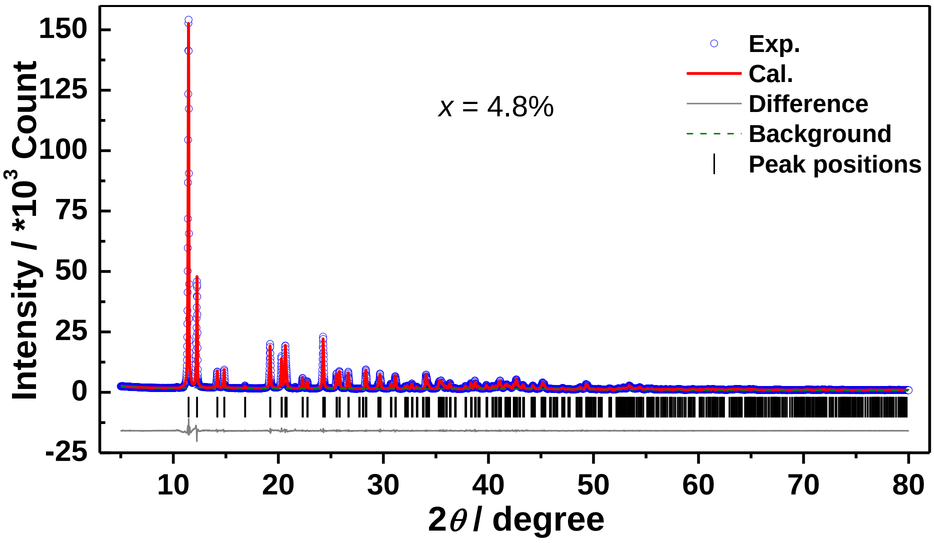
Intensity / *10^3 Count
2θ / degree
x = 4.8%
Exp.
Cal.
Difference
Background
Peak positions

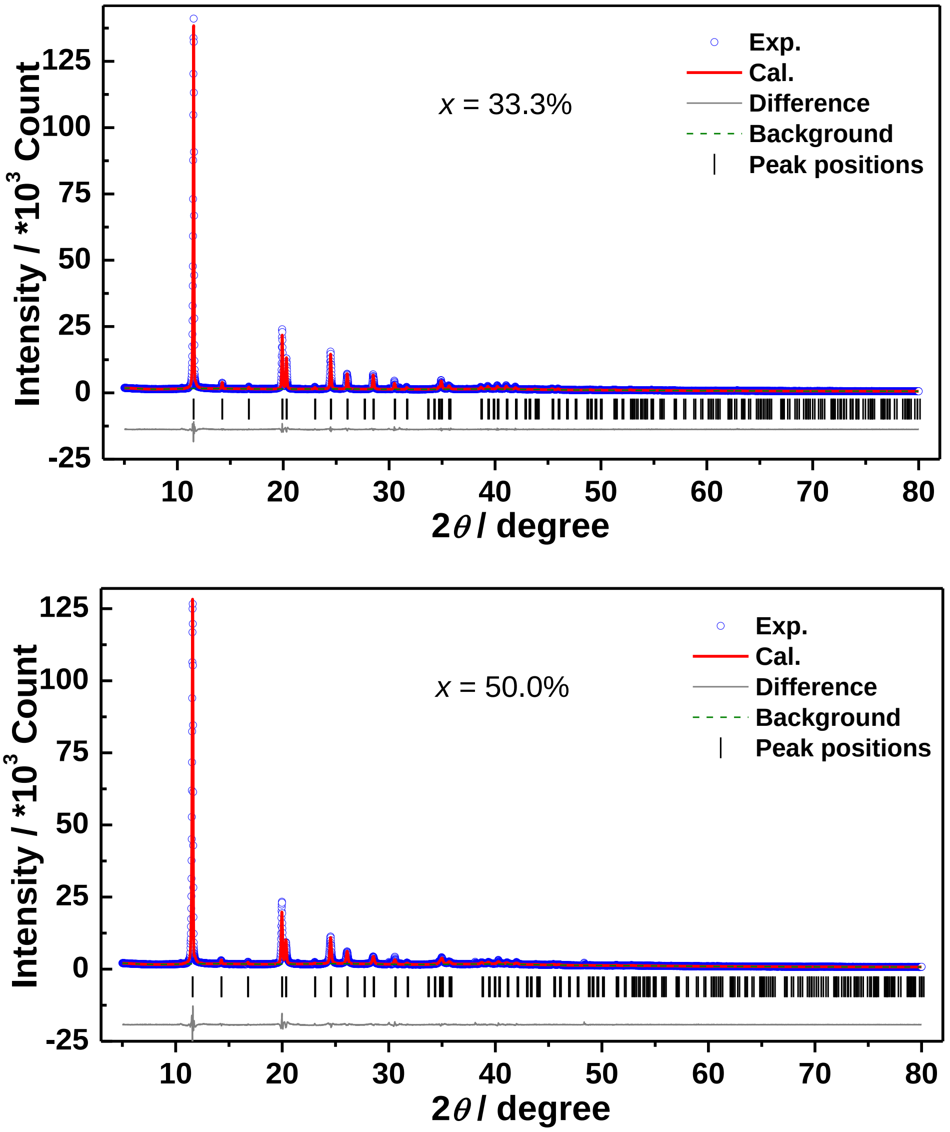

Supplementary Fig. 16 Pawley refinement on the PXRD pattern of solid solutions {$\mathbf{1}_{1-x}\mathbf{2}_x$} at room temperature. For display details, see the caption of Supplementary Fig. 12.

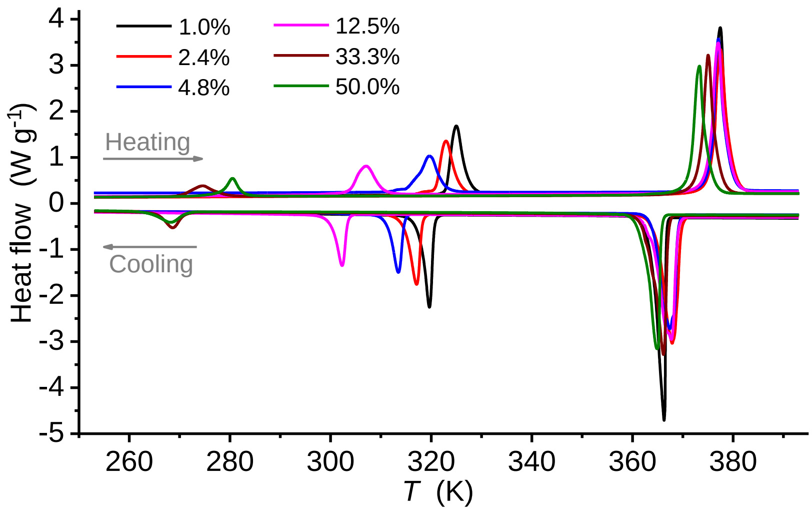

Supplementary Fig. 17 DSC measurements for the solid solutions {$\mathbf{1}_{1-x}\mathbf{2}_x$} recorded on a heating−cooling cycle. The mixing ratio (x) of CP **2** ranges from 1.0% to 50.0%,

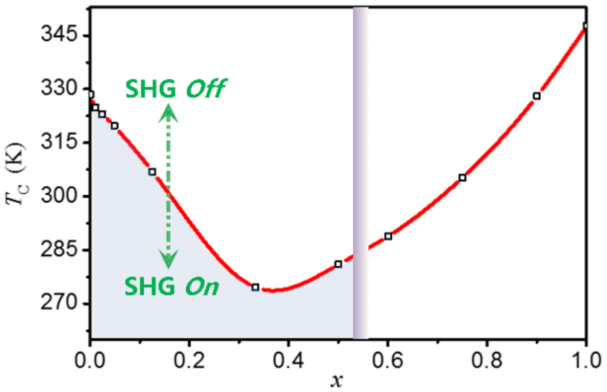

Supplementary Fig. 18 Solid-solid phase transition temperatures (upon heating) as a function of *x* in the solid solutions {$\mathbf{1}_{1-x}\mathbf{2}_x$}. The SHG active area of the solid solutions is shaded in light blue.
